# weIMPUTE: a user-friendly web-based genotype imputation platform

**DOI:** 10.3389/fgene.2025.1532464

**Published:** 2025-03-17

**Authors:** Mingliang Li, Zhuo Li, Defu Liu, Qi Li, Xiaodong Hu, Jun Yu, Jian Lin, Chunguang Bi, Guanshi Ye, Helong Yu, You Tang

**Affiliations:** ^1^ Electrical and Information Engineering College, Jilin Agricultural Science and Technology University, Jilin, China; ^2^ College of Information and Control Engineering, Jilin Institute of Chemical Technology, Jilin, China; ^3^ College of Information Technology, Jilin Agricultural University, Changchun, China; ^4^ Guangzhou College of Technology and Business School of Engineering, Guangzhou, Guangdong, China

**Keywords:** imputation, phasing, GUI, genotype, web based

## Abstract

**Background:**

Genotype imputation is a critical preprocessing step in genome-wide association studies (GWAS), enhancing statistical power for detecting associated single nucleotide polymorphisms (SNPs) by increasing marker size.

**Results:**

In response to the needs of researchers seeking user-friendly graphical tools for imputation without requiring informatics or computer expertise, we have developed weIMPUTE, a web-based imputation graphical user interface (GUI). Unlike existing genotype imputation software, weIMPUTE supports multiple imputation software, including SHAPEIT, Eagle, Minimac4, Beagle, and IMPUTE2, while encompassing the entire workflow, from quality control to data format conversion. This comprehensive platform enables both novices and experienced users to readily perform imputation tasks. For reference genotype data owners, weIMPUTE can be installed on a server or workstation, facilitating web-based imputation services without data sharing.

**Conclusion:**

weIMPUTE represents a versatile imputation solution for researchers across various fields, offering the flexibility to create personalized imputation servers on different operating systems.

## Introduction

The advent of high-throughput sequencing has revolutionized research, significantly advancing fields like genome-wide association studies (GWAS) and genome selection (GS). However, a key challenge remains in efficiently imputing low-density datasets to high-density ones. To fully harness the potential of whole genome sequencing and enhance statistical power in GWAS, as well as reduce GS costs in animal and plant breeding, a user-friendly imputation pipeline is essential. Such a pipeline should not require extensive command-line expertise or specialized knowledge of the Linux environment. Additionally, the establishment of an imputation server is a powerful means of leveraging computational resources for large datasets while offering secure, public imputation services.

Genotype imputation is a well-established method, with ongoing developments that address how factors such as reference panel composition, sample size, and population structure influence accuracy (Marchini and Howie, 2010; Ye et al., 2019; Huang et al., 2012; Vergara et al., 2018; Browning and Browning, 2016). Marchini and Howie (Marchini and Howie, 2010) provide a thorough review of statistical approaches for genotype imputation, including both Bayesian and frequentist methods. They highlight key applications such as boosting GWAS power, fine-mapping loci, and enabling cross-study comparisons. Other studies focus on strategies for combining internal and external reference panels in non-human (Loh et al., 2016) or admixed populations (Chen et al., 2019; Chattopadhyay et al., 2023), while also demonstrating that imputation can scale efficiently to millions of reference samples using memory-optimized models (Howie et al., 2009). Large reference datasets often require separating the phasing and imputation processes. Widely used software like Minimac4, Eagle2, Beagle5, SHAPEIT, and IMPUTE2 each specialize in different aspects of imputation or phasing (Loh et al., 2016; Das et al., 2016; Delaneau et al., 2014; Howie et al., 2009; Browning et al., 2018; Browning et al., 2021). Some, such as Eagle and Beagle, perform these tasks without relying on a reference panel, making them versatile for studies without such panels. Others, like SHAPEIT-IMPUTE2, are particularly effective with admixed populations due to their robust algorithms. While Minimac4 excels with homogeneous populations, it is also effective with diverse datasets when paired with large reference panels, as seen with the Michigan Imputation Server and TOPMed Imputation Server. Additionally, Beagle’s Java implementation offers cross-platform flexibility, whereas Eagle, SHAPEIT, and Minimac4 are primarily designed for Linux environments.

Public services like the TOPMed Imputation Server (TIS) (Taliun et al., 2021) and the Michigan Imputation Server (MIS) (Loh et al., 2016; Das et al., 2016) have greatly advanced large-scale imputation in human genetics. TIS utilizes a reference panel of over 97,000 genomes from the NHLBI TOPMed Program, providing high imputation accuracy for human populations. However, TIS has limitations, including restricted access due to data-sharing requirements, limited sample sizes, and data upload constraints. MIS, while also using robust reference data and supporting phasing tools like Eagle and Beagle, lacks a complete end-to-end workflow, such as integrated data preparation and post-imputation filtering. Other tools like Gimpute (Chen et al., 2019) and MI-System (Chattopadhyay et al., 2023) have been developed for large human datasets, but often require command-line proficiency or fail to provide comprehensive QC steps. Gimpute, for example, excels in imputation accuracy but lacks a cross-platform containerized framework, limiting its deployment flexibility. MI-System, though capable of handling large datasets, is designed for human studies and demands high computational resources, posing challenges for non-human applications.

To meet the diverse needs of researchers, we developed weIMPUTE, a flexible imputation platform supporting both pre-phasing (Eagle2 or SHAPEIT) and imputation software (Minimac4, Beagle5, and IMPUTE2). Leveraging Docker technology, weIMPUTE ensures seamless installation and operation across different operating systems. It integrates essential quality control (QC) steps, such as format validation and genome build liftover, along with post-imputation filtering, providing a comprehensive and efficient solution. Unlike public servers, weIMPUTE offers local or server-based installations, allowing users to manage large or specialized datasets—especially non-human species—without limitations due to public resource availability. The platform’s web-based interface makes it easy for researchers to handle datasets of varying sizes and population structures. Additionally, weIMPUTE supports user-defined reference panels, a crucial feature for studies involving underrepresented populations. Through extensive testing, we have verified that weIMPUTE incurs no additional computational overhead compared to standard command-line pipelines, while efficiently utilizing parallel job execution. While public services like TIS and MIS remain valuable for moderate-sized human studies, weIMPUTE extends the scope of imputation to large-scale and non-human applications, offering deeper workflow customization within a containerized, cross-platform framework that minimizes reliance on command-line expertise.

## Methods

**FIGURE 1:**
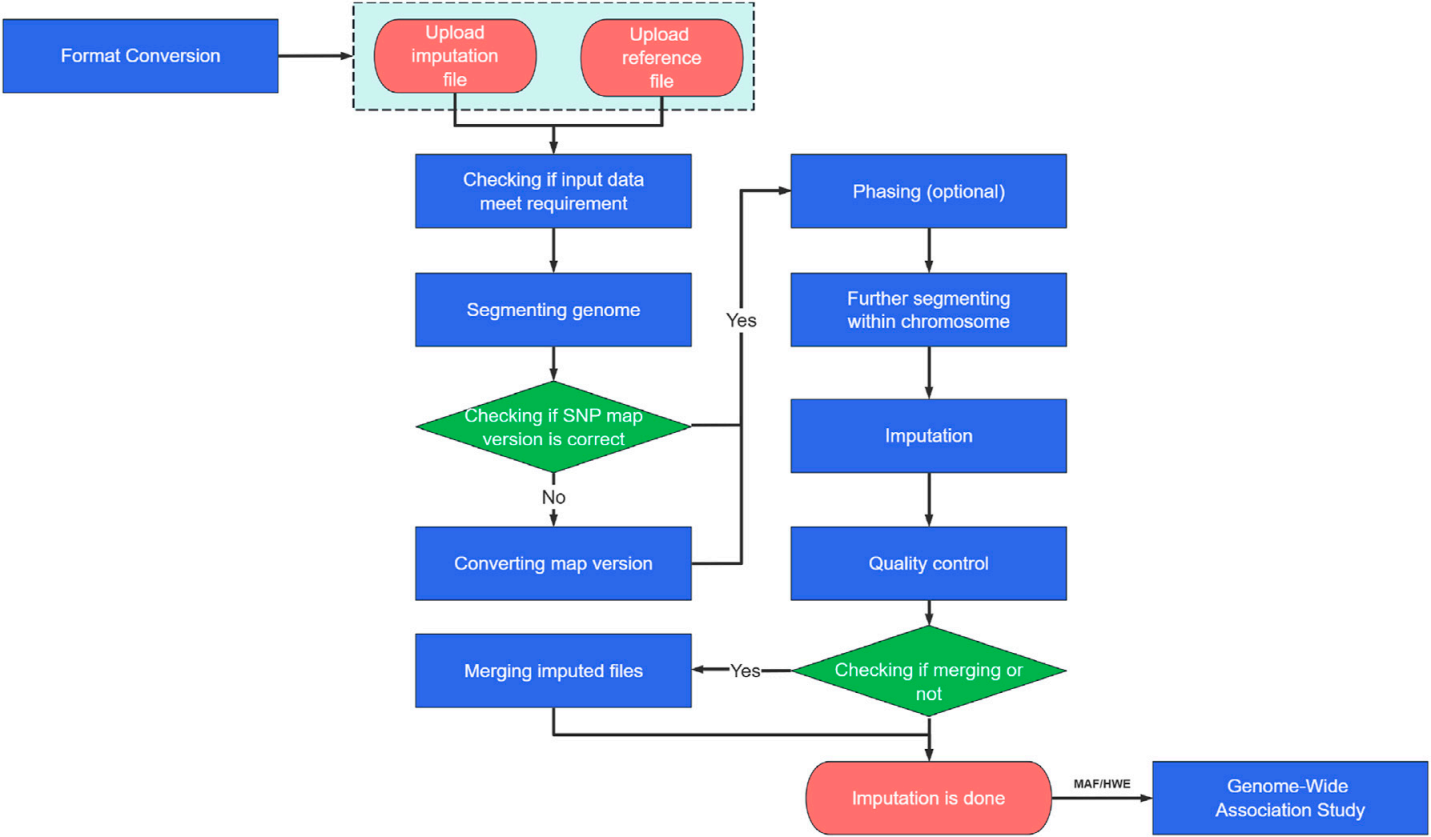
The workflow of phasing and imputation in weIMPUTE.

weIMPUTE is a comprehensive imputation platform that streamlines the entire imputation process, from data preparation and phasing to imputing and post-quality control (QC) analysis (see Figure 1). By integrating these functionalities, weIMPUTE offers automated imputation for routine tasks, such as data segmentation and command generation, while allowing manual review for tasks requiring biological relevance or dataset-specific adjustments. The platform offers multiple pipelines to attend to various imputing scenarios, such as data segmentation and parallelization, while still allowing users to perform customized tasks, including phasing and imputing large datasets.

Users can conveniently install weIMPUTE on their local machines and access it through a user-friendly website, which also ensures data security through efficient datasets management. To ensure seamless compatibility across multiple operating systems (OS), all third-party libraries and software used in weIMPUTE have been packaged using Docker, eliminating the need for additional environment configuration. To achieve effective implementation, all modules within weIMPUTE are written in C++ and Java.

In the subsequent sections, we will delve into the key modules of the weIMPUTE pipeline, providing insights into its powerful and user-centric functionalities.

### Data preparation

weIMPUTE accepts VCF format as both input and output for imputation. For Minimac4, which requires M3VCF format for the reference panel, VCF data will be converted to M3VCF using Minimac3 or m3vcftools. If the reference and inference panels use different genome map versions, the Lift-Over module aligns them accordingly. Input data is automatically segmented by chromosome, and users can further divide the data within each chromosome (e.g., into 5 million bp files) for enhanced efficiency. The QC module ensures the data meets imputation requirements, such as being phased and consistent in map version and format. If a file contains too few SNPs, a warning will prompt the user to remove it from the input list. For sex chromosomes, a dedicated imputation pipeline is automatically executed.

weIMPUTE’s Docker architecture and flexible reference panel support extend beyond human genetics, allowing successful application in non-human species, such as dogs (Supplementary Material S2). This adaptability makes weIMPUTE a versatile tool for diverse genetic architectures, provided an appropriate reference panel is available.

We also include a format conversion module to accommodate alternative genomic formats (e.g., PLINK, Hapmap). This module converts these formats into VCF, streamlining workflows, especially for users with limited bioinformatics expertise. Once converted, the data undergo the same segmentation, QC checks, and lift-over procedures, ensuring consistency regardless of the initial format. Additionally, weIMPUTE supports standard VCF-based reference panels, including M3VCF formats for Minimac4, with support for both publicly available and custom user-uploaded panels.

To ensure data privacy and security, weIMPUTE’s containerized deployment isolates imputation jobs from the host environment. Users can run weIMPUTE in an offline mode on institutional servers, ensuring that genomic data need not be uploaded elsewhere. For multi-user scenarios, we recommend enabling secured web connections and instituting user authentication for full data protection.

### Task management

weIMPUTE offers a task management service that optimizes computing resources by default. Users are relieved of the burden of tuning numerous parameters for each phasing and imputation step, as the default settings are optimized based on pilot testing with varying segment sizes and thread allocations. These defaults provide efficient performance across diverse datasets, though researchers with specialized requirements are encouraged to fine-tune further. Users have the freedom to select modules and determine their running order. Within each module, if multiple input files are present, they will be automatically assigned to different threads, optimized based on available computing resources. Task status can be monitored online or offline, and the storage management module handles automatic deletion of uploaded and output data files. Administrators can set a timer to determine when these files should be deleted.

### Imputed data merging and filtering

After imputation, the post-QC module assesses the success of the imputation process. Users have the option to filter imputed variants based on imputation quality scores (*r* ^2^ value). If desired, users can use the merge module to combine the filtered segment files into a single whole chromosome file.

### Platform portability

weIMPUTE is containerized using Docker, making it cross-platform and easily accessible through a web browser on any OS that has Docker installed. For imputing large datasets, weIMPUTE can be installed on powerful computing nodes, allowing remote access through a laptop’s web browser. Users can check and modify the imputation job status at any time without the need to log into the computing node.

## Results

### Time consumption and memory usage

**FIGURE 2:**
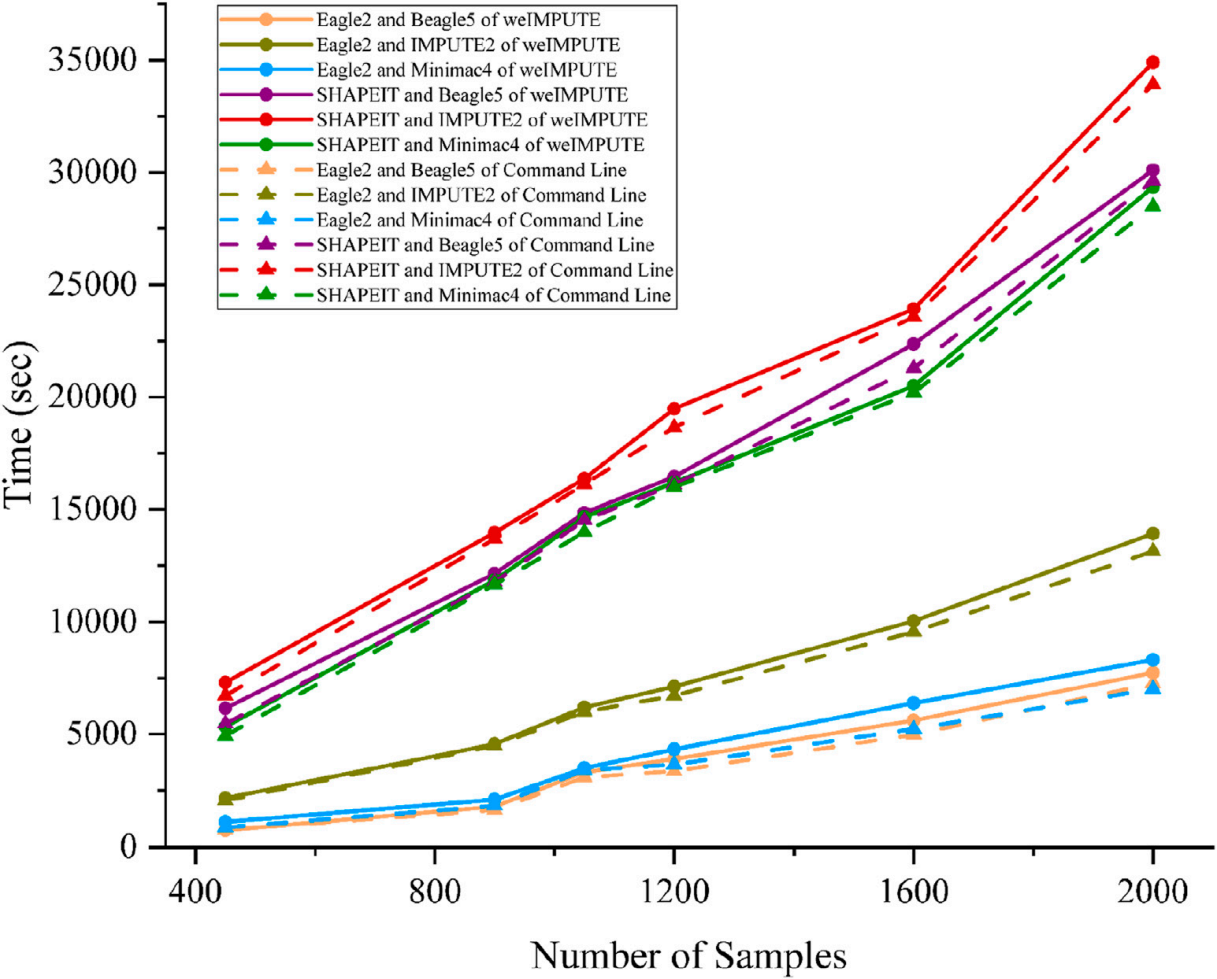
Phasing and imputation time comparison of weIMPUTE web service and command line version of various software combinations.

Given that phasing and imputation are computationally intensive tasks, excessive computing resource usage can be a concern for GUI-based imputation software. To validate weIMPUTE’s effectiveness as an imputation service, we compared its time consumption and memory usage to traditional command line operations. The results show that weIMPUTE’s GUI-based approach exhibits similar time and memory costs as command line usage (see Figure 2). Leveraging Docker as a lightweight container, weIMPUTE effectively provides imputation services with optimal resource utilization.

Benchmarks were conducted on a dataset simulated using sim-ped, consisting of six groups ranging from 450 to 2,000 samples and containing 32,503 SNPs with a genotype missing rate of 0.1%. The computational environment included a server equipped with an Intel(R) Xeon(R) Gold 6138 CPU (80 cores, 160 threads) and 440 GiB of RAM. All tasks were executed in a Docker-based environment to minimize system overhead and standardize the benchmarking process.

### Resource requirements and practical guidelines

Our simulated data is based on the real 1 KG (1,000 Genomes) data (1000 Genomes Project Consortium et al., 2015), using 1 KG individuals as founders. Specifically, we used the European (EUR) whole genome sequencing (WGS) data from the 1000 Genomes Project, which can be accessed via ftp://ftp.1000genomes.ebi.ac.uk/vol1/ftp/release/20100804/. Under single-threaded conditions, peak memory usage remained below 700 MB across Eagle, SHAPEIT, Beagle, IMPUTE2, and Minimac4 (Supplementary Material S3), with Docker’s container overhead proving negligible. By increasing the number of threads to utilize multicore environments, runtime was substantially reduced without a significant rise in memory consumption. For researchers handling datasets of this size or larger, we recommend allocating at least 2–4 GB of RAM per active thread, particularly when dealing with multiple parallel tasks on HPC clusters or cloud-based infrastructure. Moreover, segment-based workflows—splitting large chromosomes into smaller blocks (e.g., 5–10 Mb segments)—help mitigate the wall-clock time, allowing weIMPUTE to deliver efficient and robust performance while accommodating a broad spectrum of computational budgets.

weIMPUTE integrates an efficient error-handling mechanism to enhance user experience and ensure system stability. For common issues such as inconsistent chromosome naming, mismatched genome builds, or insufficient memory, the system automatically generates alerts. The user interface highlights failed tasks in the job queue and provides downloadable log files, enabling users to quickly diagnose and resolve problems. Additionally, the admin panel continuously monitors hardware resource usage, including CPU and memory allocation, allowing users to optimize performance by adjusting resource settings or task configurations. These features offer convenient troubleshooting support, particularly for novice users.

For data privacy and security, weIMPUTE’s containerized deployment isolates imputation jobs from the host environment. Users can run weIMPUTE in an offline mode on institutional servers, ensuring that genomic data need not be uploaded elsewhere. For multi-user scenarios, we recommend enabling secured web connections and instituting user authentication for full data protection.

### GWAS integration and workflow enhancement

As many researchers use imputed genotypes for downstream analyses, such as GWAS or genomic selection, we have introduced a weIMPUTE_GWAS module with enhanced features for post-imputation filtering and analysis. Before this, users can perform minor allele frequency (MAF) and Hardy-Weinberg equilibrium (HWE) filtering through the format conversion QC module. Additionally, the module provides a simplified graphical interface for widely used GWAS tools, such as GAPIT (Lipka et al., 2012; Tang et al., 2016; Wang and Zhang, 2021).

By seamlessly integrating weIMPUTE and weIMPUTE_GWAS, users can now filter variants, configure GWAS inputs (e.g., genotype files, phenotype data, covariates), execute association analyses, and visualize results—all within a single platform. This integration eliminates the need for transferring data between software packages and ensures that both post-imputation filtering and downstream analyses remain accessible and efficient, especially for researchers with limited computational expertise.

To further demonstrate the application of weIMPUTE, we present a case study (Supplementary Material S2) involving genotype imputation and genome-wide association study (GWAS) analysis in a dataset of 4,341 domestic dogs (Huang et al., 2017). The procedure includes uploading the MAP file for chromosome 1 (Supplementary Material S2), followed by haplotype phasing using EAGLE (Supplementary Material S2; Figures 2). After phasing, genotype imputation is performed using BEAGLE (Supplementary Material S2). Subsequently, quality control (QC) is applied, with a minor allele frequency (MAF) threshold set to 1% and Hardy-Weinberg equilibrium (HWE) filtering (Supplementary Material S2). After data cleaning, the GWAS analysis module is used to investigate the relationship between canine hip dysplasia (CHD) and genetic markers.

Through this process, multiple SNPs significantly associated with CHD were successfully identified, with SNP BICF2S23441691 showing a strong association across several models (Supplementary Material S2; Supplementary Table 1). The reliability of these findings is confirmed by the Manhattan plot (Supplementary Material S2) and QQ plot (Supplementary Material S2) of the output data.

### Server setup

weIMPUTE leverages Docker to enable parallel job submissions on servers or HPC clusters with multiple cores. In the updated documentation (Supplementary Material S1), we provide step-by-step instructions for installing Docker, pulling the weIMPUTE container, and customizing resource allocation. This approach ensures minimal command-line usage while allowing large datasets to be processed efficiently.

## Conclusion

weIMPUTE integrates multiple phasing tools (Eagle2, SHAPEIT) and imputation tools (Minimac4, Beagle5, IMPUTE2), each optimized for specific scenarios. Minimac4 is effective with a single, homogeneous reference panel, while IMPUTE2 supports combining multiple reference panels for admixed populations. Eagle2 is efficient for large datasets with homogeneous ancestry, while SHAPEIT is recommended for admixed datasets. For studies with over 1,000 individuals, data can be divided into chromosome chunks for parallel processing, a step automated within weIMPUTE.

While weIMPUTE automates many processes, manual checks, such as adjusting segment sizes or verifying allele concordance, remain essential, particularly for rare variants or non-human populations. The platform offers flexibility for users to select the most suitable tools and configurations for their studies, in line with established best practices such as those proposed by van Leeuwen et al. (2015) (van Leeuwen et al., 2015). The weIMPUTE_GWAS module provides basic QC functions such as MAF, HWE, and r2 filtering, but users may need additional tools for more complex QC. We recommend incorporating extra QC steps to ensure high-quality data.

In summary, weIMPUTE offers a comprehensive solution for genotype imputation, including data quality control and streamlining the process. Its cross-platform compatibility and user-friendly interface make it suitable for diverse species, as demonstrated by a case study involving 4,341 domestic dogs. By optimizing the imputation workflow, weIMPUTE addresses missing data issues and supports complex genetic studies, proving effective in handling large-scale datasets.

With weIMPUTE, researchers can seamlessly perform imputation without extensive command line knowledge. While the platform automates routine computational processes for efficiency, it also allows flexibility for manual data review and optimization to ensure accurate and biologically relevant results. This integration of functionalities and platform portability empowers researchers to harness the full potential of whole genome sequencing technology, expand sample sizes, and enhance statistical power in various research fields. weIMPUTE emerges as a valuable resource, advancing the field of genotype imputation and contributing to research advancements across different species and domains.

## Data Availability

The original contributions presented in the study are included in the article/Supplementary Material, further inquiries can be directed to the corresponding authors.
