# Supplementary material for: weIMPUTE: a user-friendly web-based genotype imputation platform
Source: Front Genet. 2025 Mar 17;16:1532464. doi: 10.3389/fgene.2025.1532464 (PMC11955643; doi:10.3389/fgene.2025.1532464)
Supplement: Supplementary file 1 [file Supplementaryfile1.docx]

**weIMPUTE Step-by-Step Guide**

**weIMPUTE** is a web-based genotype imputation platform that supports multiple genotype imputation tools such as SHAPEIT, Eagle2, Minimac4, Beagle5, and IMPUTE2. You can follow the steps below to install **weIMPUTE**, prepare input data, and use its key features.

**1. Installing weIMPUTE**

**weIMPUTE** is a web-based genotype imputation platform that supports various tools like SHAPEIT, Eagle, Minimac4, Beagle, and IMPUTE2. Follow the steps below to install **weIMPUTE** on your server.

**Step 1: Download the Installation Script**

First, download the automatic installation script for **weIMPUTE** from the following link:

- [install_weIMPUTE.sh](https://github.com/YOUTLab/weIMPUTE/blob/main/install_weIMPUTE.sh)

**Step 2: Run the Installation Script**

After downloading the script, open a terminal and execute the following commands to make the script executable and run it:

*chmod +x install_weIMPUTE.sh*

*sudo ./install_weIMPUTE.sh*

The script will automatically perform the following tasks:

- Configure the firewall and open necessary ports
- Download and load the Docker image
- Start the Docker container
- Install and configure **weIMPUTE**

This process may take a few minutes, depending on your internet speed and system performance.

**Step 3: Access the weIMPUTE Platform**

After the installation is complete, you can access the **weIMPUTE** platform using the following addresses:

- Front-end service: http://IP:9083
- Back-end management: http://IP:9085

Where IP refers to the IP address of the server where **weIMPUTE** is installed.

**Recommendation**: It is recommended to use a Linux operating system for the installation to ensure smooth operation.

**2. Data Preparation**

Before starting imputation, you need to prepare your genomic data. **weIMPUTE** supports various formats, including PLINK, Hapmap, and VCF formats.

**Step 1: Prepare the Data**

- **PLINK Format**: A common format for genotype data used in most genomic studies.
- **Hapmap Format**: Widely used for reference genotype data.
- **VCF Format**: Variant Call Format, which is the standard input format for **weIMPUTE**.

**Step 2: Data Format Conversion and Quality Control**

Use the **Format Conversion and Quality Control** tool provided by **weIMPUTE** to process your data. Common quality control steps include:

- **MAF (Minor Allele Frequency) Filtering**: Ensures high-quality input data.
- **Hardy-Weinberg Equilibrium (HWE) Check**: Identifies loci that deviate from Hardy-Weinberg equilibrium, helping to avoid errors in the imputation process.

You can refer to the detailed **Format Conversion and Quality Control** guide here: [weIMPUTE_FORMAT_Conversion.pdf](https://github.com/YOUTLab/weIMPUTE/blob/main/weIMPUTE_FORMAT%20Conversion%20Tool/weIMPUTE_FORMAT_Conversion.pdf)

**3. Running Genotype Imputation**

**weIMPUTE** supports multiple imputation tools. You can choose the tool that best suits your needs.

**Step 1: Upload the Data**

After logging into the platform, choose the option to upload your VCF format file.

**Step 2: Select the Imputation Tool**

You can choose from the following imputation tools:

- **SHAPEIT**
- **Eagle2**
- **Minimac4**
- **Beagle5**
- **IMPUTE2**

Select the appropriate tool and configure the relevant parameters.

**Step 3: Start Imputation**

Once configured, click the “Start Imputation” button to begin the imputation process. The platform will automatically initiate the imputation and you can monitor the progress via the graphical interface.

**4. Performing GWAS (Genome-Wide Association Study)**

In addition to genotype imputation, **weIMPUTE** also integrates a GWAS analysis tool. Follow the steps below to perform GWAS analysis.

**Step 1: Upload GWAS Data**

In the **weIMPUTE** platform's GWAS interface, select the option to upload your GWAS data file. The platform supports common GWAS data formats.

**Step 2: Set Analysis Parameters**

Choose the analysis method (e.g., GAPIT) and set the necessary parameters. Make sure your data and parameter settings are correct to ensure accurate analysis.

**Step 3: Run GWAS Analysis**

Click the “Start Analysis” button to run the GWAS analysis. The system will process the data and generate the results.

**Step 4: View and Visualize Results**

Once the analysis is complete, the platform will provide tools for visualizing the GWAS results, helping you to interpret and understand the findings.

**5. Management and Monitoring**

As an administrator, you can use the back-end management interface to perform the following actions:

- **User Management**: View and manage registered users.
- **Server Management**: Monitor the status of the server and manage the task queue.

Access the back-end management interface: http://IP:9085

**6. Access Example Files and Resources**

- **Example Files**: You can test the platform's functionality by uploading small demo files available within the platform.
- **Documentation**: Detailed guides and documentation are available in the GitHub repository, covering installation, data preparation, imputation, and GWAS analysis.

**Resource Links**:

- [Quick Start.pdf](https://github.com/YOUTLab/weIMPUTE/blob/main/Quick%20Start.pdf)
- [Local Installation.pdf](https://github.com/YOUTLab/weIMPUTE/blob/main/Local%20Installation.pdf)
- [weIMPUTE_FORMAT_Conversion.pdf](https://github.com/YOUTLab/weIMPUTE/blob/main/weIMPUTE_FORMAT%20Conversion%20Tool/weIMPUTE_FORMAT_Conversion.pdf)
- [weIMPUTE_GWAS.pdf](https://github.com/YOUTLab/weIMPUTE/blob/main/weIMPUTE_GWAS/weIMPUTE_GWAS.pdf)

**7. References and Support**

If you encounter any issues during usage, you can contact the author through the following:

- **Email**: tangyou9000@163.coms

Alternatively, refer to the [**FAQ**](https://github.com/YOUTLab/weIMPUTE) section in the GitHub repository for further assistance.
