# Supplementary material for: weIMPUTE: a user-friendly web-based genotype imputation platform
Source: Front Genet. 2025 Mar 17;16:1532464. doi: 10.3389/fgene.2025.1532464 (PMC11955643; doi:10.3389/fgene.2025.1532464)
Supplement: Supplementary file 2 [file Supplementaryfile3.docx]

Table 1 Peak Memory Usage (in Kb) for Various Tools

| Sample | Eagle | Shapeit | Beagle | Impute | Minimac |
| --- | --- | --- | --- | --- | --- |
| 450 | 16036 | 27153 | 139586 | 429496 | 12914 |
| 900 | 16696 | 35477 | 225485 | 471649 | 14454 |
| 1050 | 16852 | 50462 | 234631 | 503583 | 15467 |
| 1200 | 17008 | 67863 | 246960 | 524917 | 16886 |
| 1600 | 17568 | 83521 | 280467 | 561345 | 17056 |
| 2000 | 18346 | 100136 | 332859 | 657142 | 17452 |
