# Supplementary material for: weIMPUTE: a user-friendly web-based genotype imputation platform
Source: Front Genet. 2025 Mar 17;16:1532464. doi: 10.3389/fgene.2025.1532464 (PMC11955643; doi:10.3389/fgene.2025.1532464)
Supplement: Supplementary file 3 [file Supplementaryfile2.docx]

**Case Study: Genotype Imputation and GWAS Analysis in Domestic Dogs Using weIMPUTE**

This case study demonstrates the application of weIMPUTE for genotype imputation and subsequent genome-wide association study (GWAS) analysis in a dataset involving 4341 domestic dogs. The study investigates the relationship between canine hip dysplasia (CHD) and genetic markers, utilizing imputation to handle missing genotype data for 920 individuals with available phenotype data. The imputation process was performed using a reference panel of 3421 dogs without CHD phenotype data.

**1. Data Preparation and MAP File Upload**

To begin the imputation process, the MAP file for chromosome 1 of the dog was uploaded to the backend of weIMPUTE. The MAP file is essential for both genotype imputation and haplotype phasing. The user first selects “File upload,” then uploads the MAP file for chromosome 1 of the domestic dog, as shown in **Figure 1**. Additionally, the MAP file needed for BEAGLE (used in later steps) is also uploaded, following the required format as per the respective software documentation.


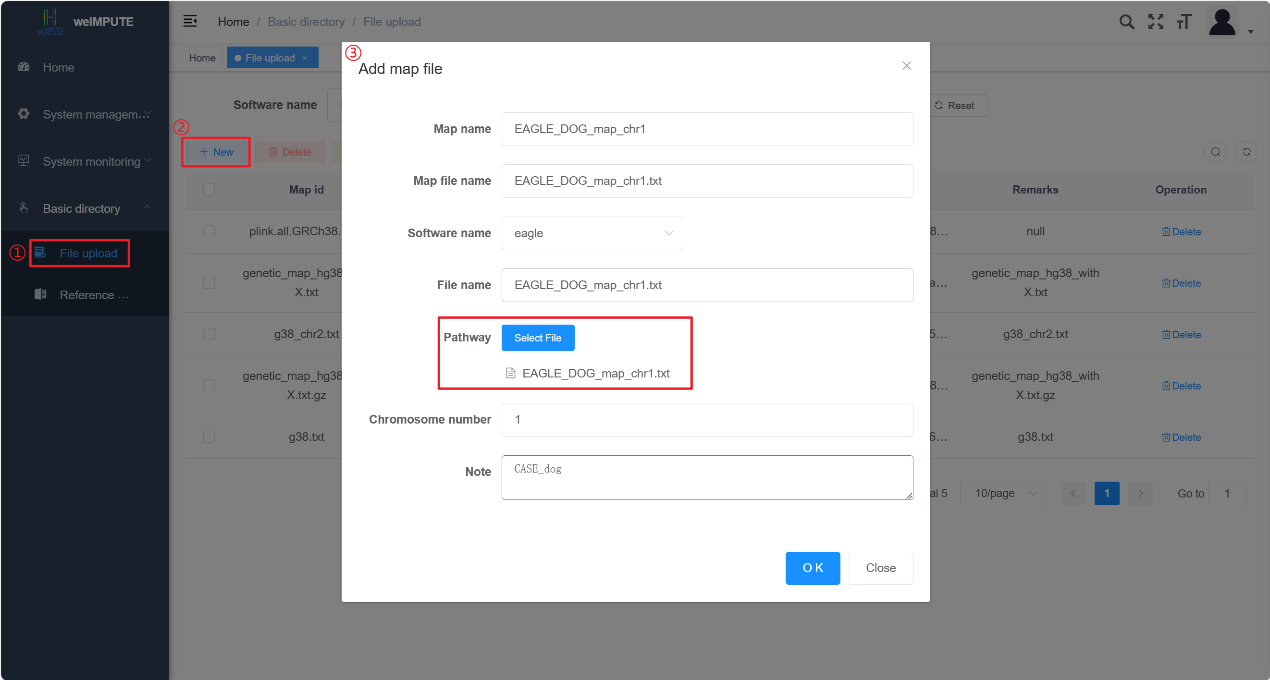


Figure 1 MAP File Upload Interface Example

**2. Haplotype Phasing**

Once the reference panel data and target genotypes are prepared, users move to the Haplotype Phasing Program in the frontend interface. In this step, the EAGLE+BEAGLE software is selected, and the imputation file (with 10% missing genotypes) is uploaded, along with the reference panel data. Given that this case pertains to a non-human species, we bypass the "chromosome type" and "lifover conversion" steps typically used for human data and proceed directly to the haplotype phasing interface.

For haplotype phasing, the EAGLE MAP file is selected, and the user clicks "Advanced setup" to adjust parameters (Figure 2).


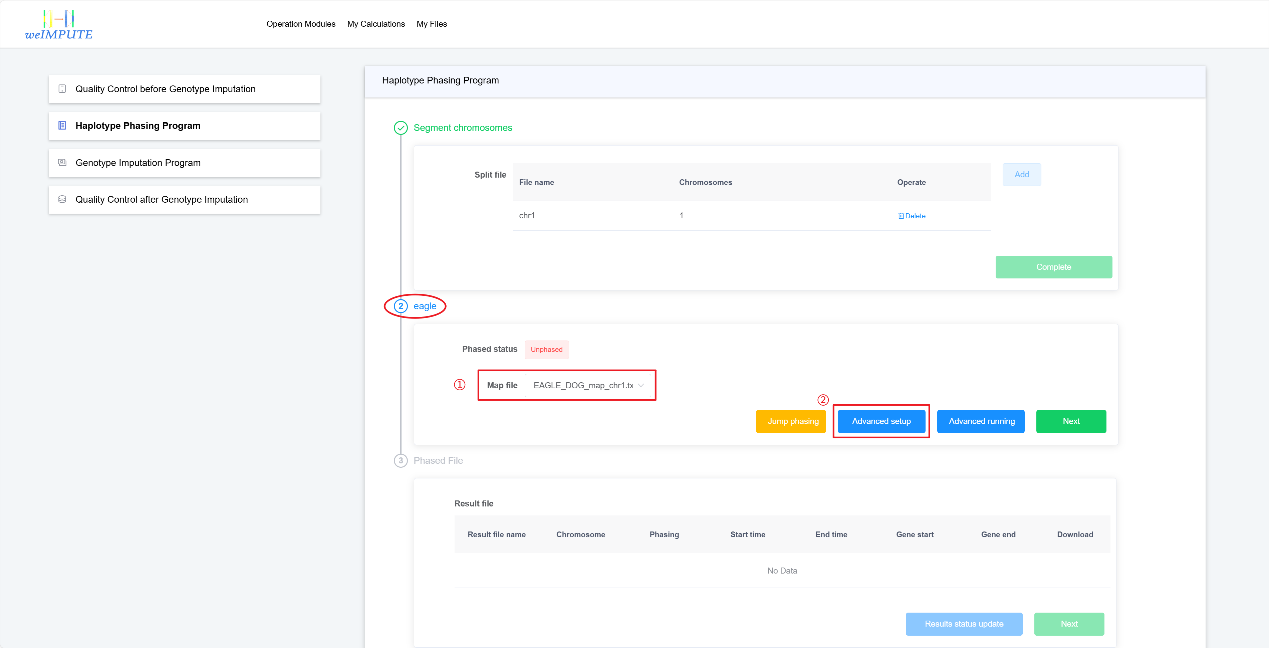


Figure 2 Haplotype Phasing Advanced Setup

Specifically, the “--noImpMissing” parameter is enabled to ensure only phasing is performed, not genotype imputation. After clicking "Advanced running," the phasing process begins (Figure 3).


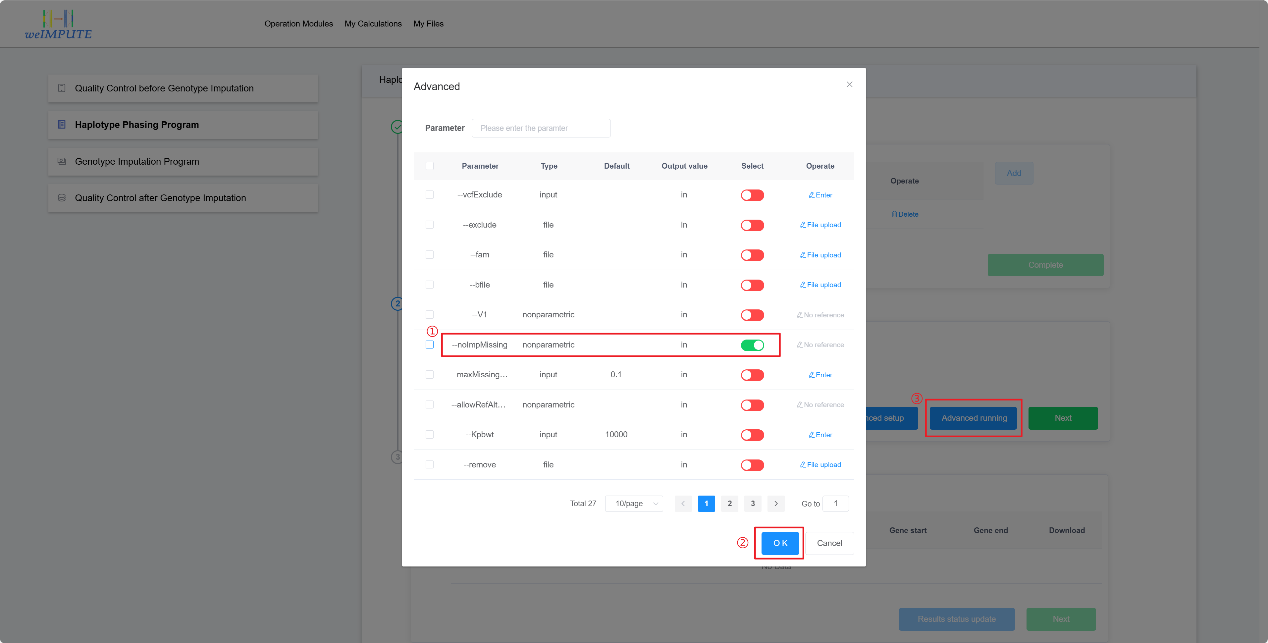


Figure 3 Haplotype Phasing Parameter Configuration

The time required for this step depends on the dataset size and machine specifications. Upon completion, users can download and inspect the resulting phased haplotypes and associated records.

**3. Genotype Imputation**

With the phasing complete, the next step is genotype imputation using the BEAGLE software. The user selects the previously uploaded BEAGLE MAP file and configures the desired parameters, including thread count and window size, based on server performance. Once settings are confirmed, clicking "Next" initiates the genotype imputation process (Figure 4).


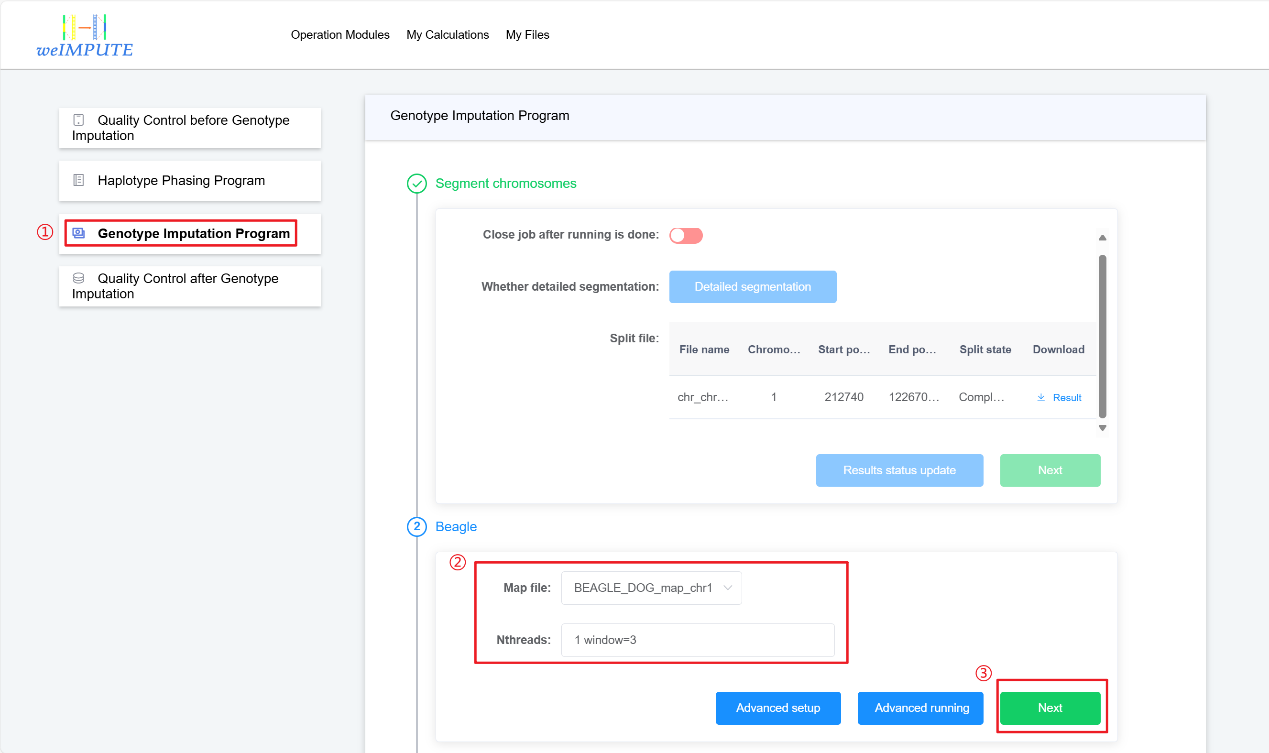


Figure 4 Genotype Imputation Configuration and Process Initiation

After the process finishes, the imputed genotypes, along with log files, are available for download.

**4. Quality Control (QC)**

Post-imputation, quality control is performed to ensure the validity of the imputed genotypes (Figure 5). The VCF file format is chosen for this step, and the imputed genotype data is uploaded for QC. Specific filters are applied: the minor allele frequency (MAF) threshold is set to exclude variants with MAF <1%, and Hardy-Weinberg equilibrium (HWE) p-value filtering is applied with a threshold of p < 1e-6. After QC, a cleaned VCF file (output.vcf.gz) is generated, ready for further analysis.


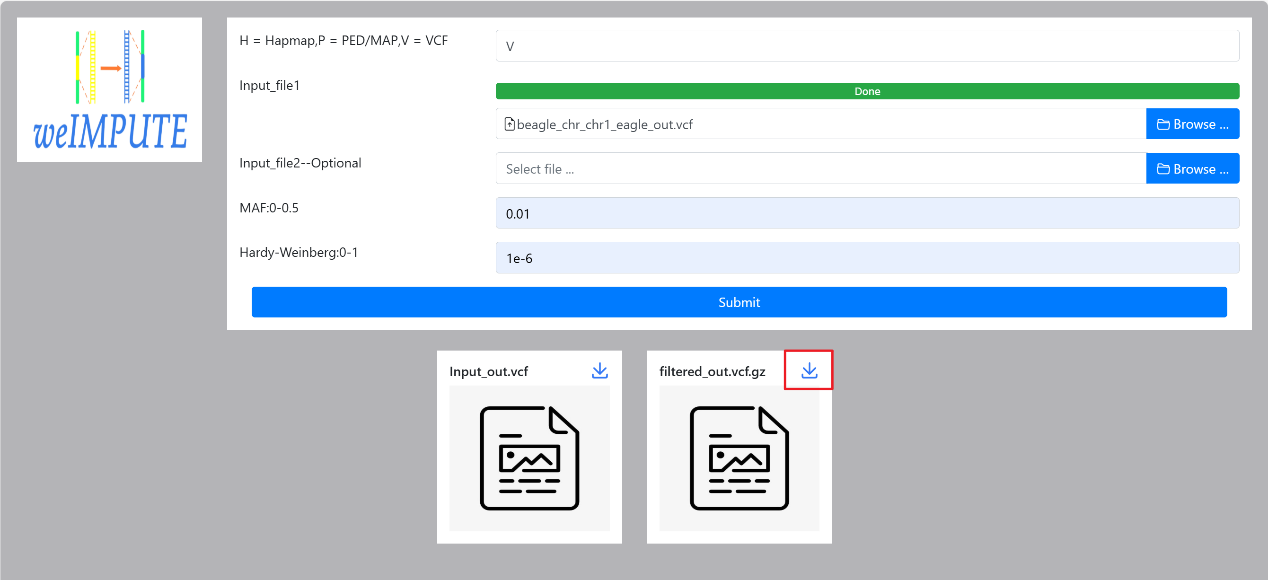


Figure 5 Quality Control Process Initiation

**5. GWAS Analysis**

The final step is the GWAS analysis. The cleaned VCF file from the QC step is uploaded, followed by the phenotype file for CHD. The phenotype file format follows the guidelines from the GAPIT software documentation. Various statistical models (e.g., GLM, FarmCPU, BLINK) are chosen to perform the association analysis. After processing, the GWAS results are displayed, and significant SNPs are identified.


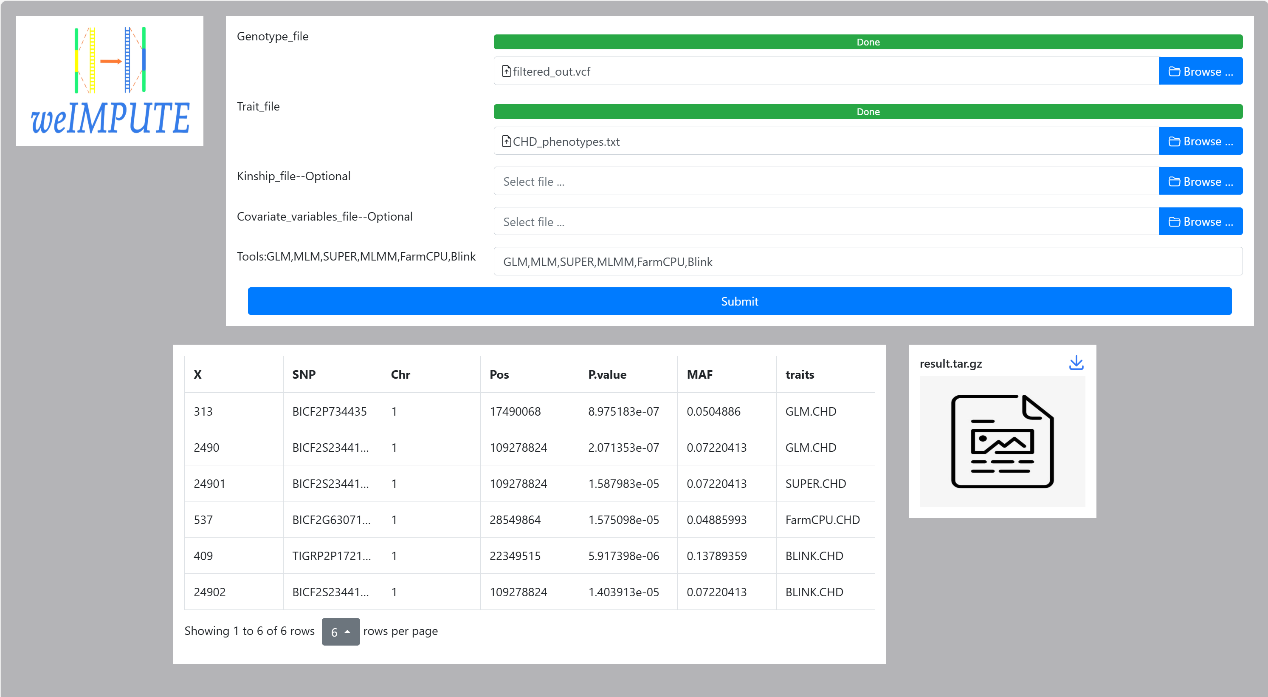


Figure 6 GWAS Analysis Workflow

**GWAS Results:**

- A table (Table 1) summarizes significant SNPs from the GWAS analysis, including SNP ID, chromosomal position, p-value, MAF, and the model used. Notably, SNP *BICF2S23441691* on chromosome 1 exhibits strong associations with CHD across multiple models.
- Figure 7 shows a Manhattan plot for the SNP-CHD associations, with SNPs located on chromosome 1 forming a "mountain-like" pattern, indicating strong genetic signals related to CHD.
- Figure 8 presents a QQ plot, which compares observed p-values with expected distributions. The close alignment of observed values with the expected line suggests minimal inflation of type I error, with slight deviations in the lower p-value region.

Table 1 Summary of Significant SNPs from GWAS Analysis

|  | SNP | Chr | Pos | P.value | MAF | traits |
| --- | --- | --- | --- | --- | --- | --- |
| 313 | BICF2P734435 | 1 | 17490068 | 8.98E-07 | 0.050489 | GLM.CHD |
| 2490 | BICF2S23441691 | 1 | 1.09E+08 | 2.07E-07 | 0.072204 | GLM.CHD |
| 24901 | BICF2S23441691 | 1 | 1.09E+08 | 1.59E-05 | 0.072204 | SUPER.CHD |
| 537 | BICF2G630715837 | 1 | 28549864 | 1.58E-05 | 0.04886 | FarmCPU.CHD |
| 409 | TIGRP2P17211_rs8916416 | 1 | 22349515 | 5.92E-06 | 0.137894 | BLINK.CHD |
| 24902 | BICF2S23441691 | 1 | 1.09E+08 | 1.40E-05 | 0.072204 | BLINK.CHD |


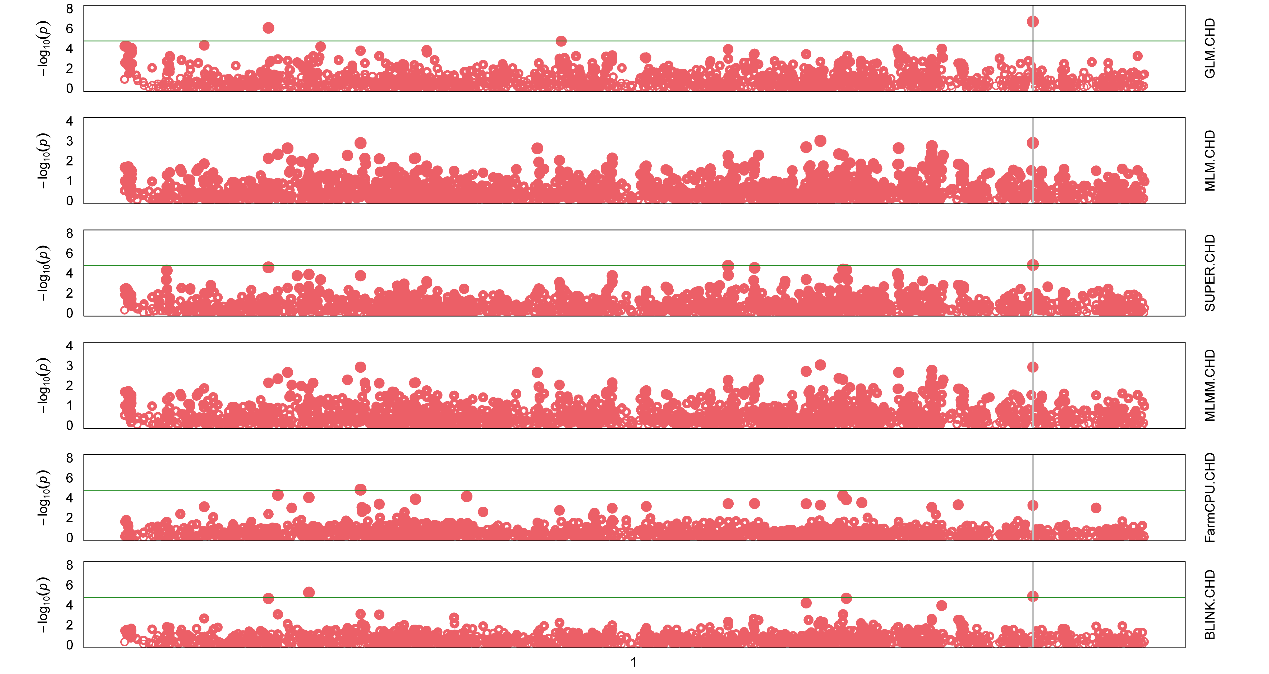


Figure 7 Manhattan Plot of SNP-CHD Associations


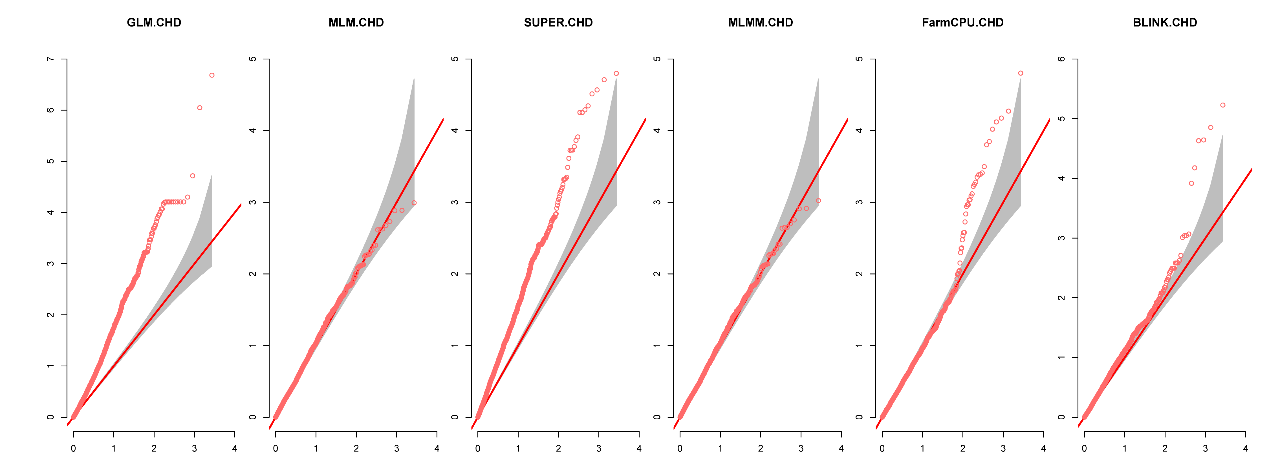


Figure 8 QQ Plot of GWAS p-values

This case study provides a clear example of how weIMPUTE can be effectively used for genotype imputation and subsequent GWAS analysis in non-human species, such as dogs, demonstrating its utility for complex genetic studies in non-human species.
